# Enhanced recovery after surgery (ERAS) program for lumbar spine fusion

**DOI:** 10.1186/s13741-019-0114-2

**Published:** 2019-05-28

**Authors:** Justin Smith, Stephen Probst, Colleen Calandra, Raphael Davis, Kentaro Sugimoto, Lizhou Nie, Tong J. Gan, Elliott Bennett-Guerrero

**Affiliations:** 1 Department of Anesthesiology, Stony Brook University Medical Center, 101 Nicolls Rd, Stony Brook, NY 11794 USA; 2 Department of Neurosurgery, Stony Brook University Medical Center, 101 Nicolls Rd, Stony Brook, NY 11794 USA; 3 Department of Applied Mathematics and Statistics, Stony Brook University, 100 Nicolls Rd, Stony Brook, NY 11794 USA

**Keywords:** ERAS, Lumbar fusion, QI spine, Spine surgery

## Abstract

**Background:**

There is a paucity of literature regarding the implementation of enhanced recovery after surgery (ERAS) protocols for open lumbar spine fusions. We implemented an ERAS program for 1–2-level lumbar spine fusion surgery and identified areas that might benefit from perioperative interventions to improve patient satisfaction and outcomes.

**Methods:**

This institutionally approved quality improvement (QI) ERAS program for lumbar spine fusion was designed for all neurosurgical patients 18 years and older scheduled for 1 or 2 level primary lumbar fusions. The ERAS bundle contained elements such as multimodal analgesia including preoperative oral acetaminophen and gabapentin, postoperative early mobilization and physical therapy, and a prophylactic multimodal antiemetic regimen to decrease postoperative nausea and vomiting. No fluid management or hemodynamic parameters were included. Pre-ERAS and post-ERAS data were compared with regard to potential confounders, compliance with the ERAS bundle, and postoperative outcomes.

**Results:**

A total of 230 patients were included from October 2013 to May 2017. The pre-ERAS phase consisted of 123 patients, 11 patients during the transition period, and 96 serving as post-ERAS patients. The pre-ERAS and post-ERAS groups had comparable demographics and comorbidities. Compliance with preoperative and intraoperative medication interventions was relatively good (~ 80%). Compliance with postoperative elements such as early physical therapy, early mobilization, and early removal of the urinary catheter was poor with no significant improvement in post-ERAS patients. There was no significant change in the amount of short-acting opioids used, but there was a decrease in the use of long-acting opioids in the post-ERAS phase (14.6 to 5.2%, *p* = 0.025). Post-ERAS patients required fewer rescue antiemetic medications in the recovery room compared to pre-ERAS patients (40 to 24%). There was no significant difference in postoperative pain scores or hospital length of stay between the two groups.

**Conclusions:**

Implementing an ERAS bundle for 1–2-level lumbar fusion had minimal effect in decreasing length of stay, but a significant decrease in postoperative opioid and rescue antiemetic use. This ERAS bundle showed mixed results likely secondary to poor ERAS protocol compliance. Going forward, this QI project will look to improve post-operative ERAS implementation to improve patient outcomes.

## Introduction

Back pain and spinal disorders are one of the most commonly encountered medical problems facing the healthcare system. Approximately two thirds of the population will suffer from low back pain (LBP) at some point in their lifetime, and it is estimated that the USA spends over $100 billion annually in direct and indirect costs related to LBP (Deyo and Weinstein 2001; Dagenais et al. 2008). A lumbar spinal fusion may improve LBP and help many improve their quality of life. While outcomes after spinal fusion are generally good, many patients experience adverse events such as superficial and deep wound infections, deep vein thrombosis, pseudarthrosis, urinary tract infections, transient ischemic attacks, and continued pain following surgery (Proietti et al. 2013).

In recent years, “fast track” surgery or enhanced recovery bundles have been developed in many surgical specialties to decrease hospital length of stay (LOS) and decrease perioperative morbidity. Kehlet first introduced the enhanced recovery model in 1997 as a multimodal, evidence-based plan to improve patient care in the perioperative period (Kehlet 1997). Since then, many ERAS strategies have shown the effectiveness of enhanced recovery bundles in improving patient outcomes. These ERAS bundles have been used successfully in colorectal surgery, radical cystectomies, major pelvic surgery, and pancreaticoduodenectomies to name a few (Gustafsson et al. 2013; Daneshmand et al. 2014; Nygren et al. 2013; Lassen et al. 2013). To date, there have been very few reports of the implementation of ERAS bundles that focused on improving patient outcomes in open lumbar fusions and spinal surgery (Wainwright et al. 2016; Blackburn et al. 2016). In this study, we hypothesized that the ERAS protocol would decrease: case cancelations, postoperative nausea and vomiting (PONV), length of stay, postoperative pain, and postoperative narcotic use.

## Methods

### ERAS protocol development and implementation

This ERAS QI protocol was implemented at Stony Brook University Hospital in Stony Brook, New York, after receiving institutional approval. The program included neurosurgical patients 18 years and older planned to undergo a 1 or 2 level lumbar spinal fusion. This QI project excluded patients who were pregnant, age < 18, or planned for a revision of a previous fusion.

The departments of neuroanesthesia and neurosurgery at our institution worked together to review neurosurgery spinal fusion cases to identify interventions that would address unmet postoperative patient care goals. The protocol was divided into preoperative, intraoperative, and postoperative interventions. While some of the bundle elements were already common practice for lumbar fusion procedures at our institution and were included in the ERAS protocol, there was no standardized care bundle for all lumbar fusion patients. Table 1 outlines the protocol.

**Table 1:** Lumbar spinal fusion ERAS protocol

Stage	Location	Action
Preoperative	Neurosurgical clinic visit	▪ 1–2-level lumbar fusion patients identified to be included in the project
		▪ Neurosurgery Spine Booking Checklist completed and surgery scheduled with the comment “lumbar fusion ERP”
		▪ Patient given information letter and materials including diabetes education and smoking cessation
		▪ Patients received a “pain questionnaire” and if any patient selects “on chronic and current benzodiazepines or opioids,” they will be considered high risk for pain and the acute pain service will be made aware
	Booking office	▪ The following appointments are made:
		§ Preoperative services
		§ OR for surgery
		§ Postoperative wound check (14 days) and surgical (30 days) follow-up
		▪ A surgical packet will be sent to the patient with the following:
		§ Cover letter explaining the contents and what to expect at POS and PSA
		§ Instructions for taking or stopping medications
		§ Directions to pre-operative services and ambulatory surgery unit
		§ Postoperative discharge instructions
		§ Social support services information
		§ Discharge needs assessment form—to be returned pre-operatively
		§ Pre-operative pain questionnaire-to be returned pre-operatively
		▪ Assistant will add the lumbar fusion ERP checklist to the surgical packet that is sent to pre-operative services
	Preoperative services	▪ History and physical-required
		▪ Anesthesia consult-required and to be performed by an anesthesiologist, qualified MD, or physician extender
		▪ Required testing includes T&S, CBC, PT/PTT, INR, UA, and for diagnosed diabetic patients HgbA1C
		▪ HgbA1c of ≥ 9 will postpone the surgical date by at least 2 months and will be re-evaluated prior to rebooking
		▪ Incentive spirometry, OSA and CPAP education, NPRS education
		▪ Pre-operative antibiotic ordered—Ancef 2 g (3 g if > 120 kg), Clindamycin 900 mg, or Vancomycin 15 mg/kg
Perioperative	Ambulatory surgery unit	▪ If identified as a “high-risk pain” the “pain liaison” will visit the patient prior to OR
		*▪ Acetaminophen 975 mg PO, gabapentin 900 mg PO prior to OR*
		*▪ High-risk patients administer 40 mg aprepitant for PONV*
	OR for surgery	▪ Antibiotics will be dosed and given less than 1 h prior to incision and re-dosed as appropriate
		*▪ All patients regardless of diabetic status will receive dexamethasone 8 mg IVP*
		*▪ High-risk patients with known chronic pain may receive ketamine 30 mg with induction*
		▪ Follow intra-op protocol as prescribed including hourly attending anesthesia to neurosurgeon communication
		§ Should include progress of surgery, fluid status, hemodynamics, pressure point evaluations, EBL
	PACU	▪ Assess patient temperature
		▪ *All patients receive PCA and methocarbamol 1500 mg PO or IV*
		▪ Pain liaison will visit the patient if identified as “high-risk pain”
Postoperative	Floor	▪ Nursing staff will notify neurosurgery if there are any medically necessary deviations from the protocol.
		▪ PT/SW/NS to meet Monday–Friday to review patients’ discharge progress
	POD#0	▪ All patients will receive stool softeners and laxatives delineated in the power plan
		▪ Diet as tolerated
		▪ Continue IVF’s until tolerating good oral intake
		▪ Reinforce incentive spirometry
	POD#1	*▪ Discontinue Foley catheter at 0600*
		*▪ Celecoxib 200 mg Q12H PO, gabapentin 300 mg Q8H PO, and acetaminophen 975 mg Q6H PO to be continued for 1 week*
		▪ Acute pain assessment for the transition to oral medications
		*▪ Mobilize out of bed and reinforce incentive spirometry*
		*▪ PT evaluation for rehabilitation needs*
		*▪ SW/Case management evaluation for support services and discharge planning*
	POD#2	▪ If appropriate discontinue surgical drain and continue to mobilize
		▪ Discharge if appropriate
Follow Up	Neurosurgical Clinic Visit	▪ Follow-up phone call post-discharge day 1
		▪ Wound check visit 2 weeks after discharge
		▪ Regularly scheduled follow-up at 1 month, 3 months, 6 months, and 1 year

The data in italics are the important interventions for measured outcomes

*POS* preoperative services, *PSA* presurgical admission, *OSA* obstructive sleep apnea, *CPAP* continuous positive airway pressure, *NPRS* numeric pain rating scale, *PONV* postoperative nausea/vomiting, *IVP* IV push, *EBL* estimated blood loss, *PCA* patient-controlled analgesia, *PT* physical therapy, *SW* social work, *NS* neurosurgery

Among the gaps of the existing care protocol, patient communication was observed to be an important issue to address since some patients were receiving inconsistent recommendations concerning medications to be taken on the day of surgery, and expectations following surgery varied between patients. It was also found that patients with chronic pain were not identified prior to surgery, and there was a need for better pain management postoperatively for all patients. Earlier involvement of physical therapy and social work was another area for improvement to decrease delays in mobilization and discharge. To address these issues, our goals included: improving preoperative patient education, decreasing case cancelations, decreasing hospital LOS, decreasing PONV, decreasing postoperative pain, and decreasing postoperative opioid use.

To improve patient communication, a standardized education packet was given to patients in the neurosurgical clinic prior to surgery. This included information about the surgery, expectations, support services, management of diabetes, and smoking cessation among other things. To decrease case cancelations, the neurosurgery office administrative staff had an increased role in communicating with the surgeon and operating room staff to ensure proper scheduling of cases and to notify care providers of the need to adhere to the ERAS protocol. At preoperative services, education was reinforced and patients underwent a laboratory workup, history and physical prior to surgery. As a means to decrease PONV, all patients were scheduled to receive dexamethasone 8 mg IV after induction of anesthesia, and for patients with more than 2 risk factors (Apfel et al. 2012), oral aprepitant 40 mg was added in the preoperative holding area. Patients were routinely given intraoperative ondansetron 4 mg IV for PONV prophylaxis. The protocol did not include intraoperative fluid or hemodynamic parameters. To decrease postoperative pain and opioid use, a multimodal analgesia regimen was included (Mathiesen et al. 2013). On the day of surgery, the patient was reassessed by the anesthesia care provider and given acetaminophen 975 mg PO and gabapentin 900 mg PO in the preoperative holding area. All patients were given an intake form during their initial consultation with the neurosurgeon. Patients who marked that they were “on chronic and current benzodiazepines or opioids” on this form were considered high risk for pain. The acute pain service was to be made aware of all high-risk pain patients, and they were to receive ketamine 30 mg IV with the induction of anesthesia (Loftus et al. 2010). There were no specific guidelines for intraoperative opioid use. Prior to leaving the recovery room a fentanyl, morphine, or hydromorphone, PCA was started, and methocarbamol 1500 mg IV or PO was given to manage pain. Patients also received a non-opioid regimen for 7 days including celecoxib 200 mg Q12H PO, gabapentin 300 mg Q8H PO, and acetaminophen 975 mg Q6H PO (Doleman et al. 2015). To decrease hospital LOS, the postoperative interventions included early physical therapy (PT) and social work involvement on postoperative day 1 with early mobilization. Each patient was contacted the day after discharge by phone and then seen in the office for a 2-week wound check visit. Following the wound check visit, each patient was scheduled for regular follow-up at 3-, 6-, and 12-month intervals.

### Data collection

The pre-ERAS patients (historical control group) were identified through the electronic medical record (EMR) and included patients who underwent 1–2-level lumbar fusion surgery between October 23, 2013, and September 9, 2015. A transition phase after the ERAS protocol was started lasted from September 10, 2015, to November 5, 2015. During this transition phase, staff and physicians were educated on the protocol and became familiar with its steps to improve compliance. Regular meetings of all ERAS team leads and members were held. The entire ERAS protocol was made available in the EMR to be used as a reference when needed, and reminders were integrated into the intraoperative anesthesia EMR. After the transition phase, post-ERAS patients underwent surgery between November 9, 2015, and May 3, 2017.

All preoperative, intraoperative, and postoperative data were collected from the EMR for both the pre-ERAS and ERAS groups and entered into a database. We followed patients for up to 1 year from their surgical date. This report includes data collected from the preoperative period through patients’ first postoperative visit within 30 days of discharge from the hospital (Table 5).

### Prespecified quality metrics

Prespecified quality metrics included the number of case cancelations, the incidence of postoperative nausea and vomiting (PONV), opioid usage, postoperative pain, and length of stay. The protocol planned to use rescheduled cases as a measure to track case cancelations. Length of stay was determined based on a patient’s admission time and discharge time as recorded in the EMR for the encounter number that correlated to the surgical admission for lumbar fusion. PCA use and all other analgesic medications used were recorded for postoperative day 0 through postoperative day 3, at which time it was expected that a majority of patients would be discharged. Medication use at each postoperative visit was also recorded. Patient pain was measured based on an 11-point numerical pain rating scale (NPRS) in which patients rate their pain ranging from 0 (no pain) to 10 (worst imaginable pain). The NPRS has been shown to be effective at showing pain improvement in patients with low back pain when the NPRS shows a difference of greater than 2 points (Childs et al. 2005). The NPRS was measured prior to surgery, each day during their hospital stay, and at each postoperative clinic visit. During this project, each NPRS measurement was multiplied by 10 to create a pain score range of 0 (no pain) to 100 (worst imaginable pain) to simplify the analysis of pain scores.

### Statistical methods

A statistician (co-author LN) performed all statistical analyses. Categorical variables were computed using the Monte Carlo simulations of exact *p* values from Pearson’s chi-squared tests. For continuous variables, *p* values were computed using the Wilcoxon rank sum test if normality checks using the Shapiro-Wilk test failed. Otherwise, two-sample *t* tests were used. If variances from pre-ERAS and post-ERAS were found to be unequal using the *F* test, then the Satterthwaite two-sample *t* test was used. Otherwise, the pooled two-sample *t* test was used. As this was a QI project, there was no formal hypothesis testing, nor any formal sample size calculation.

## Results

### Demographics

Overall, 230 eligible surgical patients were included. There were 123 patients in the pre-ERAS group (23-month period), 11 patients in the transition phase (2-month period), and 96 patients in the post-ERAS group (18-month period). The pre-ERAS and post-ERAS group patients were not significantly different with regard to potential confounders such as demographics and comorbidities except for the rate of obstructive sleep apnea (4.2% in the post-ERAS group and 12.9% in the pre-ERAS group, see Table 2).

**Table 2:** Demographic and co-morbidities

	Pre-ERAS (*n* = 123)	Post-ERAS (*n* = 96)	*p value
Number of patients	123	96	
Gender, # and % male	53 (43.1%)	48 (50.0%)	0.3395
Age	60.3 (12.9)	61.3 (13.3)	0.7308
BMI	29.7 (5.5)	29.7 (4.8)	0.7783
Coronary artery disease, #(%)	15 (12.2%)	10 (10.4%)	0.8286
Hypertension, #(%)	68 (55.3%)	50 (52.1%)	0.6715
Asthma, #(%)	13 (10.6%)	7 (7.3%)	0.4834
Chronic obstructive pulmonary disease, #(%)	8 (6.5%)	7 (7.3%)	1.0000
Diabetes mellitus—non-insulin dependent, #(%)	9 (7.3%)	15 (15.6%)	0.0794
Diabetes mellitus—insulin dependent, #(%)	4 (3.3%)	2 (2.1%)	0.7009
History of cerebrovascular accident, #(%)	4 (3.3%)	5 (5.2%)	0.5096
Anxiety, #(%)	22 (17.9%)	17 (17.7%)	1.0000
Depression, #(%)	18 (14.6%)	10 (10.4%)	0.4078
Kidney disease, #(%)	10 (8.1%)	4 (4.2%)	0.2775
Liver disease, #(%)		2 (2.1%)	0.1926
Obstructive sleep apnea, #(%)	16 (13.0%)	4 (4.2%)	0.0326
Alcohol abuse, #(%)	2 (1.6%)		0.5024
Tobacco abuse, #(%)			
• Yes, NOT within the last 6 months	39 (31.7%)	41 (42.7%)	0.1199
• Yes, within the last 6 months	25 (20.3%)	15 (15.6%)	0.3850
Substance abuse, #(%)			
• Yes, NOT within the last 6 months	2 (1.6%)	4 (4.2%)	0.4307
• Yes, within the last 6 months	3 (2.4%)	2 (2.1%)	1.0000
ASA PS class ≥ 3	63 (51.2%)	50 (52.1%)	1.0000
Non-surgical treatments utilized			
• Physical therapy	51 (41.5%)	23 (24.0%)	0.0104
• Acupuncture	17 (13.8%)	8 (8.3%)	0.2928
• Chiropractic	27 (22.0%)	14 (14.6%)	0.2222
• Epidural or facet injections	78 (63.4%)	51 (53.1%)	0.1310
Preoperative pain medication (last 30 days)			
• NSAIDs	76 (61.8%)	48 (50.0%)	0.0992
• Opioids, short-acting (immediate-release)	57 (46.3%)	47 (49.0%)	0.7852
• Opioids, long-acting (extended-release, e.g., OxyContin, MSContin, Methadone)	4 (3.3%)	2 (2.1%)	0.7021
• Anticonvulsants	30 (24.4%)	29 (30.2%)	0.3720
• Antidepressants	31 (25.2%)	29 (30.2%)	0.4464
• Benzodiazepines	18 (14.6%)	12 (12.5%)	0.6964
• Muscle relaxants	24 (19.5%)	11 (11.5%)	0.1350
• Acetaminophen	42 (34.1%)	34 (35.4%)	0.8826

Perioperative characteristics were also similar between the two groups (Table 3). Postoperative mobility and complications were not statistically different between the pre-ERAS and post-ERAS groups.

**Table 3:** Perioperative data

	Pre-ERAS (*n* = 123)	Post-ERAS (*n* = 96)	*p value
Preoperative pain score (NPRS)	63.7 (24.7)	69.5 (22.3)	0.0969
Pre-operative medications given orally on the day of surgery prior to OR			
• Gabapentin 900 mg	4 (3.3%)	75 (78.1%)	<.0001
• Acetaminophen 975 mg	3 (2.4%)	78 (81.3%)	<.0001
• Aprepitant 40 mg	1 (0.8%)	2 (2.1%)	0.5786
Number of levels fused			
• 1	71 (57.7%)	54 (56.3%)	0.8906
• 2	52 (42.3%)	42 (43.8%)	0.8879
Estimated blood loss > 300 mL	24 (19.5%)	33 (34.4%)	0.0152
Blood products given in OR, #(%)	1 (0.8%)	1 (1.0%)	1.0000
Colloid given in OR	13 (10.6%)	8 (8.3%)	0.6480
Volume of crystalloid (mL)	2070.9 (736.2)	1761.5 (739.2)	0.0003
Drain placed	118 (95.9%)	96 (100.0%)	0.0720
Dexamethasone 8 mg IV intraoperative	6 (4.9%)	26 (27.1%)	<.0001
Ketamine 30 mg on induction	2 (1.6%)	5 (5.2%)	0.2497
Duration of surgery (min)	184.7 (85.4)	212.2 (140.4)	0.3093
Intraoperative dural tear	4 (3.3%)	1 (1.0%)	0.3959

### Protocol compliance

Compliance with the ERAS bundle was mixed (Table 4). On the day of surgery, post-ERAS patients received oral gabapentin and acetaminophen with 78% and 81% compliance rates, respectively. Dexamethasone administered after induction of general anesthesia increased from 4.8% of pre-ERAS patients to 27% of post-ERAS patients (*p* < 0.0001, Table 3). There was only a small increase in the number of patients receiving ketamine in the post-ERAS group, which was not significant. All patients were to receive 1500 mg methocarbamol and a PCA in the recovery room. The rate of methocarbamol use improved in the post-ERAS group (44% of pre-ERAS versus 62% in post ERAS groups, *p* = 0.0137). Prior to ERAS, 93% of patients were already receiving a PCA so the slight increase to 94.8% was not surprising.

**Table 4:** Postoperative data

	Pre-ERAS (*n* = 123)	Post-ERAS (*n* = 96)	*p value
PACU temperature on arrival	35.8 (5.1)	36.5 (0.4)	0.4443
Antiemetic given in PACU	49 (39.8%)	23 (24.0%)	0.0125
Methocarbamol 1500 mg given in PACU	54 (43.9%)	59 (61.5%)	0.0137
NPRS before PACU discharge	50.0 (22.3)	43.7 (23.9)	0.0925
Total patients with PCA	115 (93.5%)	91 (94.8%)	0.7803
• Morphine, # (% of total PCA)	56 (45.5%)	38 (39.6%)	0.4209
• Hydromorphone, # (% of total PCA)	55 (44.7%)	51 (53.1%)	0.2236
• Fentanyl, # (% of total PCA)	5 (4.1%)	2 (2.1%)	0.4634
PCA duration≥ 24 h	9 (7.3%)		0.0116
Urinary catheter duration ≥ 24 h	42 (34.1%)	30 (31.3%)	0.6565
Day of surgery medications given			
• NSAIDs	4 (3.3%)	10 (10.4%)	0.0533
• Opioids, short-acting (immediate-release)	123 (100.0%)	95 (99.0%)	0.4343
• Opioids, long-acting (extended-release, e.g., OxyContin, MSContin, Methadone)	6 (4.9%)		0.0345
• Anticonvulsants	22 (17.9%)	55 (57.3%)	<.0001
• Antidepressants	8 (6.5%)	5 (5.2%)	0.7788
• Benzodiazepines	19 (15.4%)	16 (16.7%)	0.8518
• Muscle relaxants	97 (78.9%)	83 (86.5%)	0.1603
• Acetaminophen	62 (50.4%)	45 (46.9%)	0.6868
Postoperative day #1 NPRS pain scores			
• Minimum pain score	10.8 (16.0)	15.3 (17.0)	0.0249
• Maximum pain score	75.0 (19.4)	75.8 (18.4)	0.791
Postoperative day #1 medications given			
• NSAIDs	6 (4.9%)	11 (11.5%)	0.0777
• Opioids, short-acting (immediate-release)	119 (96.7%)	92 (95.8%)	1.0000
• Opioids, long-acting (extended-release, e.g., OxyContin, MSContin, Methadone)	23 (18.7%)	5 (5.2%)	0.0028
• Anticonvulsants	30 (24.4%)	71 (74.0%)	<.0001
• Antidepressants	20 (16.3%)	10 (10.4%)	0.2312
• Benzodiazepines	35 (28.5%)	33 (34.4%)	0.3779
• Muscle relaxants	113 (91.9%)	87 (90.6%)	0.8096
• Acetaminophen	98 (79.7%)	86 (89.6%)	0.0620
Postoperative day #2 NPRS pain scores			
• Minimum pain score	6.9 (12.1)	15.3 (18.9)	0.0003
• Maximum pain score	73.5 (18.9)	74.8 (19.7)	0.5559
Postoperative day #2 medications given (for patients not yet discharged)			
• NSAIDs	7 (5.7%)	9 (9.4%)	0.4352
• Opioids, short-acting (immediate-release)	111 (90.2%)	83 (86.5%)	0.4063
• Opioids, long acting (extended-release, e.g., OxyContin, MSContin, Methadone)	28 (22.8%)	5 (5.2%)	0.0002
• Anticonvulsants	30 (24.4%)	66 (68.8%)	<.0001
• Antidepressants	22 (17.9%)	10 (10.4%)	0.1311
• Benzodiazepines	34 (27.6%)	29 (30.2%)	0.7643
• Muscle relaxants	112 (91.1%)	86 (89.6%)	0.8108
• Acetaminophen	94 (76.4%)	82 (85.4%)	0.1236
Postoperative day #3 NPRS pain scores			
• Minimum pain score	8.9 (14.0)	15.6 (19.1)	0.0158
• Maximum pain score	69.1 (22.6)	68.0 (21.4)	0.499
Postoperative day #3 medications given (for patients not yet discharged)			
• NSAIDs	7 (5.7%)	7 (7.3%)	0.7846
• Opioids, short-acting (immediate-release)	91 (74.0%)	65 (67.7%)	0.3713
• Opioids, long-acting (extended-release, e.g., OxyContin, MSContin, Methadone)	22 (17.9%)	4 (4.2%)	0.0020
• Anticonvulsants	29 (23.6%)	52 (54.2%)	0.0001
• Antidepressants	16 (13.0%)	6 (6.3%)	0.1114
• Benzodiazepines	24 (19.5%)	18 (18.8%)	1.0000
• Muscle relaxants	97 (78.9%)	66 (68.8%)	0.1208
• Acetaminophen	63 (51.2%)	54 (56.3%)	0.4919
Postoperative day patient out of bed	2.2 (0.4)	2.3 (0.6)	0.4102
Postoperative day patient seen by PT	2.2 (0.4)	2.2 (0.5)	0.6943
Postoperative complications			
• Reintubation	2 (1.6%)	1 (1.0%)	1.0000
• MI			
• Death			
• Rapid response		2 (2.1%)	0.1928
• Other		4 (4.2%)	0.0355
Length of stay (hours)	96.2 (32.0)	92.3 (36.9)	0.1372

### Outcomes

After the implementation of ERAS, we observed a statistically significant (*p* = 0.0125) decrease in the administration of rescue antiemetics in the recovery room for nausea (40% pre-ERAS versus 24% post-ERAS) (Table 4). With regard to opioid administration, in the pre-ERAS group, 7% of patients required a PCA after 24 h, whereas this decreased to 0% post-ERAS. Long-acting opioids included OxyContin, MSContin, Methadone, or any other opioids that are designated as an extended-release. Short-acting opioids were defined as any opioid not included as an extended-release or long-acting opioid. No significant differences were observed in the number of patients requiring short-acting opioids postoperatively or at the first postoperative visit. However, we observed a benefit with regard to long-acting opioids with lower use at the first postoperative visit after implementation of ERAS (14.6% pre-ERAS vs. 5.2% post-ERAS, *p* = 0.0253, see Table 5). The percentage of patients receiving anticonvulsants at discharge increased from 22 to 67% in the pre-ERAS and post-ERAS groups, respectively. This increase was expected since it reflects the use of gabapentin postoperatively as part of the ERAS bundle. The length of stay between the pre-ERAS and post-ERAS groups decreased by 5 h from 96 to 92 h; however, this did not reach statistical significance (*p* = 0.1372). Postoperative pain scores were similar in both groups. The NPRS at the post-discharge follow-up visit was also not statistically different between the two groups.

**Table 5:** Postoperative follow-up within 30 days of discharge

	Pre-ERAS (*n* = 123)	Post-ERAS (*n* = 96)	*p value
Patient contacted 1 day after discharge	4 (3.3%)	9 (9.4%)	0.0803
Pain at postoperative wound visit (NPRS)	45.6 (25.3)	48.9 (26.0)	0.3575
Medications used at postoperative wound visit			
• NSAIDs	32 (26.0%)	24 (25.0%)	0.8728
• Opioids, short-acting (immediate-release)	100 (81.3%)	80 (83.3%)	0.7247
• Opioids, long-acting (extended-release, e.g., OxyContin, MSContin, Methadone)	18 (14.6%)	5 (5.2%)	0.0253
• Anticonvulsants	27 (22.0%)	64 (66.7%)	< .0001
• Antidepressants	25 (20.3%)	22 (22.9%)	0.7416
• Benzodiazepines	27 (22.0%)	23 (24.0%)	0.7449
• Muscle relaxants	97 (78.9%)	63 (65.6%)	0.0348
• Acetaminophen	49 (39.8%)	48 (50.0%)	0.1675
Signs of infection present	2 (1.6%)	2 (2.1%)	1.0000

## Discussion

Our results did show a small improvement in the incidence of PONV and a decrease in the use of long-acting opioid, but no differences in postoperative pain, short-term opioid use, and length of stay were found.

Patient care often lags behind the most recent evidence-based practice recommendations. The ERAS movement was started to improve efficiency, reduce morbidity, improve patient experience, and decrease cost. Our spine ERAS QI protocol was implemented to improve post-operative patient care and surgical outcomes similar to what has been done with ERAS in other surgical specialties.

There are few reports of ERAS in spine surgery, with most publications focusing on the implications and feasibility of implementing a spine ERAS program (Wainwright et al. 2016; Blackburn et al. 2016; Ali et al. 2018). Blackburn et al. implemented an ERAS bundle for all elective spine surgeries including some lumbar fusions (Blackburn et al. 2016), and the other spinal ERAS projects have investigated endoscopic lumbar fusions and correction of scoliosis (Wang et al. 2017; Gornitzky et al. 2016; Muhly et al. 2016). Based on these other reports, our ERAS protocol was more pragmatic and focus on improving postoperative outcomes by decreasing case cancelations, incidence of PONV, postoperative pain, postoperative opioid usage, and length of hospital stay.

By focusing on improved communication between the clinic staff, surgeons, anesthesiologists, and operating room scheduling staff, we hoped to decrease delays and case cancelations. We attempted to measure case cancelations by determining which lumbar fusions had been rescheduled, but we found that this was unreliable due to case cancelations that occurred for unrelated reasons such as illness and other patient factors. It should be mentioned that the neuroanesthesiologists and neurosurgeons remarked that after the ERAS implementation, patients were better prepared on the day of surgery, and there were fewer delays due to problems with surgical consents, waiting for labs, retrieving missing preoperative evaluations, and medication histories.

Pain control was another focus of the ERAS protocol. Our ERAS protocol included oral non-opioid medications preoperatively to help reduce opioid needs postoperatively. The acute pain service was to be made aware of all high-risk pain patients, and they were to receive ketamine 30 mg IV with the induction of anesthesia. All patients were to receive a PCA postoperatively, and 1500 mg of methocarbamol in the PACU for early pain control. Gabapentin, celecoxib, and acetaminophen were also added to the postoperative pain regimen which is reflected in the increased number of patients on anti-convulsant medications in the postoperative period. A multimodal approach to pain treatment has been shown to decrease postoperative opioid use and decreased time to mobilization in spine surgery (Mathiesen et al. 2013). The Post-ERAS patients showed decreased postoperative opioid use with significantly fewer patients using long-acting opioids postoperatively, and none of the post-ERAS patients required a PCA after 24 h. The ERAS interventions did not change the number of patients using short-acting opioids in the postoperative period, but it should be recognized that this was a qualitative measurement and the morphine equivalents of all opioids used were not collected as part of the data. Despite the decrease in long-acting opioid use, there was no difference in pain scores between the pre-ERAS and post-ERAS groups.

PONV is often a problem in patients following surgery. In addition to the regular antiemetic regimen that is given to most patients including intraoperative ondansetron, the ERAS protocol included dexamethasone 8 mg after induction and oral aprepitant for high-risk patients preoperatively. Despite a small increase of 4 to 27% compliance between the pre- and post-ERAS patients receiving dexamethasone intraoperatively and only 2 patients receiving aprepitant in the post ERAS group, there was still a significant decrease in the patients requiring a rescue antiemetic in the recovery room. With improved compliance, this may improve further in the future.

Decreasing LOS helps to reduce costs and is an important outcome measure in many enhanced recovery protocols (Gustafsson et al. 2013; Nygren et al. 2013; Lassen et al. 2013). There are many factors that affect LOS. Preoperative comorbidities are not the sole contributor to LOS, and the most significant factors that prolong LOS are postoperative events such as bleeding, drains, late mobilization, and delayed discharge to rehabilitation facilities (Gruskay et al. 2015). To help patients get out of bed on postoperative day 1, the protocol required urinary catheters to be removed in the morning after surgery, but compliance was poor with no change between the two groups. The protocol also included early involvement of social work to identify any potential discharge needs and to have physical therapy get all patients out of bed on postoperative day 1 (Kanaan et al. 2015). Social work and physical therapy are both very busy services in our institution, and involving them early was difficult due to time constraints and limited coverage on the weekends. The average time for physical therapy to see the pre-ERAS and post-ERAS patients was similar at 2 days postoperatively. We did not observe any significant reduction in LOS, which may be related to poor compliance of protocol adherence. Hence, we were unable to determine whether the ERAS protocol elements have any significant effect on LOS.

New ERAS protocols not only focus on specific perioperative interventions by surgeons and anesthesiologists, but they have expanded to focus on preoperative patient education, building more effective team care models, improving patient satisfaction, and improved discharge planning. Our ERAS protocol focused on some of these expanded ideas to improve patient education and interdepartmental teamwork. Preoperative patient education has become an important part of improving patient care perioperatively. Educating patients about expectations postoperatively can improve postoperative patient satisfaction and decrease patient morbidities and pain scores after lumbar surgery (Archer et al. 2011). Smoking cessation is also an important issue included in the preoperative patient education. Smokers experience a higher rate of postoperative pseudarthrosis and infection after spinal fusion. Smoking cessation can help decrease these complications depending on the timing of smoking cessation (Jackson 2nd and Devine 2016). Smoking cessation was reiterated at both the neurosurgery clinic visits and preoperative services visit in addition to printed literature that patients received. Information regarding fasting guidelines and day of surgery medication use has also been an issue for some patients in the past at our institution, so education on these topics was provided to patients verbally and in printed handouts prior to surgery.

Some of the limitations of this study include a single institution, and it was a non-randomized, non-blinded project with historical patients identified from a record search of the EMR. Compliance from nursing, surgical, and anesthesia teams in following the protocol was also suboptimal which is reflected in the intervention compliance. There are many barriers to implementing ERAS protocols such as ineffective communication among team members, patient non-compliance, staff turnover with the need for continued education, and physician and staff non-compliance. This is not unique to this ERAS project and has been shown in other studies as well (Kahokehr et al. 2009; Pedziwiatr et al. 2015). Another limitation of this study was that pain medication use was measured qualitatively and not quantitatively. The use of morphine equivalents would have provided a better comparison of narcotic pain use. It is also worth mentioning that improving communication in the perioperative period made all care providers aware of the ongoing QA project and may lead to the Hawthorne effect. Providers involved in care of the ERAS patients may have been more particular about charting and the care they provided, because they were aware that data was being collected. This change in behavior by itself may result in better outcomes.

Despite the limitations, we were able to successfully implement the spine ERAS protocol at our institution with improvement in some aspects of patient outcomes. The care of the lumbar fusion patients intraoperatively has become more standardized, and perioperative teams have become more familiar with the protocol and compliance has continued to improve. This early data showing decreases in PONV and long-acting opioid use is also promising as we continue to move forward with this project. This study also demonstrates the areas where implementations are most challenging for ERAS QI projects. Future studies can focus on these areas for further compliance improvement.

## Conclusions

In our study, the ERAS protocol was associated with a decrease in the incidence of postoperative nausea, a shorter duration of opioid use and a decrease in long-acting opioid use. It also improved communication among the perioperative team and improvement in patient education preoperatively. However, it did not result in clinically significant reductions in LOS, decreased postoperative pain scores, or decreased short-acting opioid use. Moving forward, we have implemented steps and education to improve adherence to the protocol, in particular improving the timeliness of postoperative physical therapy and social work assessment of patients.

## Abbreviations

ASA PS: American Society of Anesthesiologists physical status
CPAP: Continuous positive airway pressure
EBL: Estimated blood loss
EMR: Electronic medical record
ERAS: Enhanced recovery after surgery
ERP: Enhanced recovery protocol
IV: Intravenous
IVP: Intravenous push
LBP: Low back pain
LOS: Length of stay
NPRS: Numerical pain rating scale
NS: Neurosurgery
NSAID: Non-steroidal anti-inflammatory drug
OR: Operating room
OSA: Obstructive sleep apnea
PACU: Post-anesthesia care unit
PCA: Patient-controlled analgesia
PO: Per os
PONV: Postoperative nausea and vomiting
POS: Preoperative services
PSA: Presurgical admission
PT: Physical therapy
QI: Quality improvement
SW: Social work
